# Implementation and evaluation of a mentorship program in clinical master in family medicine during the COVID-19 pandemic at the Arabian Gulf University: a longitudinal study

**DOI:** 10.1186/s12909-024-05677-8

**Published:** 2024-07-12

**Authors:** Fatema Habbash, Afif Ben Salah, Mohamed Hany Shehata, Basheer Makarem, Sadok Chlif, Amer Almarabheh, Abdelhalim Deifalla, Jaleela S. Jawad

**Affiliations:** 1 Department of Family and Community Medicine, College of Medicine and Medical Sciences, Arabian Gulf University, Manama, Bahrain; 2 Department of Family Medicine, University Medical Center King Abdullah Medical City Bahrain, Manama, Bahrain; 3 Institut Pasteur de Tunis, Tunis, Tunisia; 4 Department of Family Medicine, Helwan University, Helwan, Egypt; 5 Department of Anatomy, College of Medicine and Medical Sciences, Arabian Gulf University, Manama, Bahrain; 6 Department of Human Anatomy and Embryology, Suez Canal University, Ismailia, Egypt; 7 Ministry of Health, Manama, Kingdom of Bahrain

**Keywords:** Mentorship Program, Mixed method, Performance, Challenges, Interventions, Family medicine, Postgraduate, COVID-19, High-risk trainees

## Abstract

**Background:**

We implemented a contextualized innovative mentorship program in the Clinical Master in Family Medicine (CMFM) program established in April 2020 at Arabian Gulf University. In this paper, we describe the process of this program and derive the major challenges faced by trainees and related corrective actions and their outcomes on high-risk trainees for optimal performance.

**Methods:**

We conducted a mixed-method longitudinal study of 80 trainees, analyzing information extracted from the Moodle learning platform about five key performance indicators as well as the contents (quantitative and qualitative) of mentoring meeting reports submitted through a validated online form between 2020 and 2022. We analyzed frequencies and themes of challenges and compared trainees' performance according to time and level of risk.

**Results:**

The follow-up of all 80 trainees in two cohorts (40 for each cohort) shows that most are female (93.75%) and the mean age is 30.00 ± 2.19 years with a ratio of mentors to mentees of 1 to 5. Meetings are conducted through phone calls, virtually, and face-to-face in 62%, 29%, and 8.3% respectively. The mean number and duration of meetings are 30.88 ± 2.31 and 20.08 ± 9.50 min respectively. Time management is the most reported challenge (41.3%), followed by health, social, and psychological-related issues in 7.6%, 4.6%, and 3% respectively. We extracted four main themes related to trainees, settings of training, e-Portfolio, and the COVID-19 pandemic. The mentorship program captured 12 trainees at high risk for low academic progress (12%) of whom six graduated on time and the remaining had to repeat a few courses the following terms. The performance of the program is stable over time (mean GPA of 3.30 (SE = 0.03), versus 3.34 (SE = 0.05) for cohorts 1 and 2 in the two years respectively, (*P* = 0.33). However, it is slightly lower among high-risk trainees compared to the remaining (GPA = 3.35 (SE = 0.03) versus 3.14 (SE = 0.08), *P* = 0.043) though above the minimum of the threshold of 3 out of 4, required for the master's degree.

**Conclusion:**

The mentorship program captured the struggling trainees and permitted to implement pertinent corrective actions timely, particularly in the context of a two-year intensive CMFM program during the COVID-19 pandemic.

## Background

Mentorship is an insightful process in which guidance is ensured from an experienced and trusted advisor in a supportive relationship, that requires active participation from both the mentor and the mentee [1, 2]. The mentor role was recognized historically to be responsible for the mentees’ education, shaping their character, and supporting the overall growth and development of the individual at a critical point [3–5]. Mentoring is identified to be an important asset in academic medicine, which impacts and helps shape the careers of the future generation of healthcare providers [6]. Mentoring programs are crucial in fostering a learner-centered environment for promoting professionalism and humanistic values while maintaining a work-life balance [6, 7]. Mentors are role models who can support in tracking and supporting the individual academic and personal progress, making links over time, and helping the mentee identify areas of improvement in a safe and non-judgmental relationship [8]. Mentorship in academic medicine has also been recognized to have an important impact on personal growth and development, increased academic productivity, career guidance, job satisfaction, networking in the field of interest, and research productivity and publication [8–11]. In postgraduate medical education, formal mentorship programs were distinguished to provide an effective teaching–learning strategy that is strongly associated with passing board exams and career preparation and satisfaction [4, 6, 12, 13]. Mentored medical residents were nearly twice as likely to describe excellent career preparation and highlighted the importance of mentoring to career advancement and identity formation [13]. Early academic mentoring impacted positively career development in cardiothoracic surgery specialization as 24% of the mentored students and trainees have completed or are enrolled in higher research degrees, 18.9% were enrolled or have completed doctoral degrees, and 81% of participants have published at least one journal article [4].

The Mentoring programs established in several medical schools worldwide vary in their goals and objectives [2]. The process of implementation of these programs is adapted to fit specific institutional or target group needs [2]. While some mentoring programs are designed for medical students in all years, or at specific stages of training, others are tailored to postgraduate medical training [6, 14–16]. Medical school mentoring programs are usually based on successful initiatives at other organizations and adapted to the context and feedback from different stakeholders. Needs analysis and program piloting are rarely conducted to ensure adequate design and effectiveness before implementation [2, 17, 18]. Mentoring programs in different contexts vary in how mentors are assigned mentees, the mentor’s role, frequency, and format of meetings. While some programs allow mentees to choose their mentors, others are allocated randomly [6, 17, 19]. Interestingly, students’ peer mentoring is integrated into some initiatives to support physician mentoring [20, 21]. Mentoring meetings are often held in person, but other modes of communication, such as email and phone, are increasingly being employed [2, 22]. The frequency of meetings varies according to the aims of the specific program and meetings might take place in a clinical setting, university, or outside the workplace [2]. Mentoring activities in various programs tend to take place over a considerable period to enable the cultivation of successful mentor relationships [23, 24]. Finally, the topics covered during mentoring meetings may include diverse areas of discussion such as motivation, clinical supervision, discussion of specific mentee-selected topics, feedback, ethics, personal development plans, and career planning [25–27].

Mentoring benefits are of value to mentees, mentors, medical programs, and institutions [2]. Mentoring has been identified as fundamental to the retention and recruitment of trainees in different specialties, advancement in clinical care, as well as enhancement of research outputs and academia [2, 28, 29]. Mentoring has been reported to support the personal and professional development of students and junior doctors through constructive feedback and observing positive role models as well as helping in developing insight into subspecialty training and career guidance, enhancing self-esteem, satisfaction, and stress management [13, 27, 30].

Despite their benefits, mentoring programs might face several challenges. This is especially recognized when mentoring is informal and lacks structures and standards for consistency. Such challenges might arise when mentors are not trained and prepared for this role and lack a protected time for this function [30–32]. Furthermore, mentee engagement with mentoring can be a challenge, when students’ engagement and perceived benefits are low [2, 24, 30, 31].

The literature review about mentorship programs revealed that they rarely rely on mixed methods to grasp a comprehensive understanding of the needs of trainees and the impact of the program. Furthermore, most of these programs are not based on standardized guidelines before their implementation. The longitudinal follow-up of the effectiveness of the program and multi-level control of the smooth running is lacking.

This study builds on these previous experiences and attempts to address these gaps. It highlights the specific features of the mentoring program in The Clinical Master in Family Medicine (CMFM), at Arabian Gulf University and evaluates its effectiveness, especially during the critical period of the Coronavirus disease 2019 (COVID-19) pandemic. 

## Methodology

### Study objectives

This study aims to: i) highlight the innovative features of the mentorship program of the Clinical Master in Family Medicine (CMFM) at the Arabian Gulf University (AGU); ii) derive the major challenges faced by trainees and related corrective actions; iii) Evaluate the impact of this mentorship program (short term represented in trainees’ performance).

### Study design and population

We conducted a longitudinal study design using a mixed-method approach for data collection. The study population includes two cohorts of 80 CMFM trainees enrolled and graduated between 2020 and 2023.

### Study settings and process of the mentorship program

The CMFM program is a two-year clinically oriented postgraduate program at AGU which was established in April 2020 during the COVID-19 pandemic. The program combines different modalities of training, mainly clinical training in primary and secondary care, theoretical group activities, and quality improvement research projects. During this period, trainees are evaluated longitudinally through formative workplace-based assessments and summative end-of-each-year written (Multiple Choice Questions) and Objective Structured Clinical Examinations (OSCE). The program's intensity, implementation within the COVID-19 period, and the diversity of learning modalities within a short period required a contextualized mentorship program.

Therefore, the academic committee conceived a mentoring program after an extensive literature review based on the program's needs and desired outcomes. The program was further adapted based on program piloting and stakeholders' feedback. Through this program, we aimed to ensure real-time monitoring of professional growth and optimal academic progress and well-being of trainees during each rotation. In addition, to identify struggling trainees timely who require personalized interventions.

### Recruitment of mentors, training, and mentoring meetings process

Mentors were recruited based on their clinical experience in training in family medicine (more than five years), dedication, and motivation for the mentor role. All trainees enrolled in the CMFM program were enrolled in the mentoring program, and every five trainees were assigned a mentor by the program coordinator, however, there was flexibility in assigning mentees to specific mentors based on their request. A mentoring guide developed by the program committee was shared with mentors and mentees to provide an orientation and details about the process and outcomes of the program. Furthermore, induction workshops for both mentors and mentees were conducted to discuss details related to the mentoring program and the mentor's role. One-to-one meetings between the mentor and each trainee take place every twelve weeks but can be requested according to the mentee’s needs. For convenience and sustainability, the mentors and mentees had the option to conduct the meetings either face-to-face, virtual, or through phone calls.

As part of the continuous monitoring and evaluation of the mentorship program, continuous meetings were conducted after each phase of training with both mentors and mentees separately, to obtain feedback and highlight areas for improvement. During mentoring meetings, mentors and mentees were encouraged to reflect on and discuss achievements, feedback, and potential problems (physical, mental, or social) that could affect training. Mentors also have access to the trainees' electronic portfolio (E-portfolio) documentation summarized in a dashboard for each mentee for each rotation. The academic committee considers five Key performance indicators (KPIs) in the dashboard of high priority. They are monitored in an electronic dashboard extracted from the learning platform “Moodle”. They include attendance, daily cases encountered (coded according to the international classification of primary care), skills, and procedures performed or observed by the trainee, and participation in educational activities. In addition, the mentor is encouraged to review and discuss with the trainee detailed documentation and reflection on selected submitted clinical cases to ensure deep learning and self-confidence. By the end of the meeting, the mentor and the mentee identify the gaps and agree on a plan for the coming period. The mentor can decide if the mentee needs a meeting with the academic committee in case of a serious issue that could hinder the trainee’s personal well-being and academic progress.

**Fig. 1:**
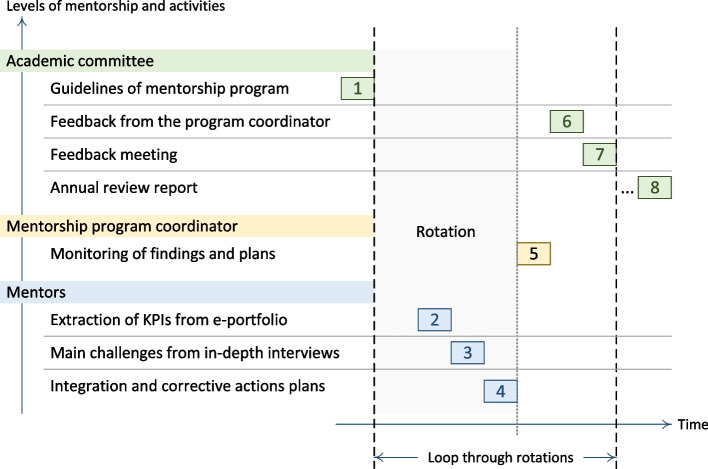
Main activities implemented by different stakeholders in the mentorship program during and after every rotation between 2020 and 2023 (activities are sequentially numbered with different colors according to mentorship level)

To ensure a standardized process of information, the program’s committee conceived a structured electronic mentoring report form on the Moodle platform to guide discussion and probe areas that need follow-up and specific interventions by the program’s committee. High-risk trainees who were identified to have sub-optimal performance or health/well-being issues are subject to more extensive mentoring by the program’s committee until resolving the identified problems. Figure 1 illustrates the multi-level framework and the cycle of the mentorship program during its implementation. The mentoring domains and related challenges are derived during every rotation at three levels: the mentors, the mentorship program coordinator, and the academic committee. At each level, specific tasks and tools are utilized to ensure a deep, comprehensive, and real-time assessment of the trainees’ progress and identify the threats. A personalized intervention plan is timely deployed and monitored at all levels until resolved.

### Data collection

All trainees are enrolled in the mentorship program and included in this study (80 trainees, 40 for each cohort). All submitted mentoring reports by all the assigned mentors between September 2020 to April 2023 were included. In addition, to findings of academic committee meetings with the trainees. We extracted the data from, the electronic mentoring report form submitted by the mentors, and academic committee reports of meetings with trainees. Information collected from the mentoring reports includes the type, frequency of meetings, duration of meetings in minutes, and dashboard KPIs generated from the E-portfolio. The latter includes attendance (a percentage), daily cases encountered (a number with access to a Word file for every case), detailed reflective cases (a number with access to a Word file for every case), skills and procedures practiced (a number with a word file for every procedure), and educational activities (a number with word file for every activity). The type of challenges (available as a list of categories: health, psychological, social, time management, others, and the recommended action plan (available as a list of categories: specific topic reading/online course, adapt learning approaches, time management, engaging in team learning activities, others). The qualitative data collected from the mentors' documentation is provided in the open-text sections and academic committee meetings’ reports. It includes details related to training progress in primary and secondary care courses, progress in quality improvement projects, challenges faced by trainees, interventions/ recommendations agreed between mentors and mentees, and corrective actions implemented by the academic committee. The thematic analysis was conducted manually following the steps of the one sheet of paper (OSOP) technique of Ziebland. We integrated the transcripts from the mentorship text file, the academic committee file, and the review report file for every trainee. Two members of the academic committee read all the transcripts for every trainee and merged them into one transcript and then we extracted themes that emerged from the data. A second iteration of reading the transcripts permitted to linking of subthemes and quotes to the most likely related theme and subthemes as illustrated in Fig. 3. Data related to mentees’ GPAs were provided by the program’s officials and the university registration unit.

### Case definitions

Cohort 1 refers to the first 40 trainees enrolled in the program in April 2020 and Cohort 2 refers to the group of 40 trainees who started the program in May 2021.

Graduation with a master's degree requires the completion of the two-year curriculum and obtaining a cumulative GPA of a minimum of 3 out of 4. Trainees who had an accumulative GPA between 2 and 3 were entitled to a Diploma degree unless they wished to repeat a certain number of courses based on the university regulations to improve their GPA to obtain the master’s degree.

A trainee is considered high risk if having one of the following situations:

1. Not able to accomplish the required level of achievement in the KPIs (related to attendance, number of documented daily cases encountered, skills and procedures, continuous medical education activities, and quality of documentation in the reflective detailed clinical cases of the E-portfolio.
2. If exposed to any health or psychosocial threat that prevents normal progress in the training.
3. Having low academic performance in formative and/or summative assessments.

### Statistical analysis plan

Statistical Analysis relies on a mixed method approach to calculate proportions and means for continuous quantitative variables, as well as thematic analysis for the qualitative textual information. The cohort and time effect were assessed using the T-test or the Mann–Whitney U test to account for the lack of normality (based on the significance of Kolmogorov–Smirnov). All quantitative analyses are performed using SPSS V28. The thematic analysis was conducted manually following the one sheet of paper (OSOP) technique of Ziebland [33].

## Results

### Participants’ characteristics

All trainees are enrolled in the mentorship program and included in this study (80 trainees, 40 for each cohort). Most of the trainees were female (93.75%) and the mean age was 30.00 ± 2.19 years. This reflects the national statistics of primary care physicians in The Kingdom of Bahrain where most family physicians are female (77.7%). A total of 16 mentors were involved with a ratio of 5 trainees per mentor. The monitoring meetings were conducted either through phone calls (62%), virtually (29.7%), or face-to-face (8.3%). The mean number of meetings during the evaluation period (20 months) was 3.88 ± 2.31 and the mean duration for the meetings was 20.08 min ± 9.50. Data related to the mentorship program indicators are presented in Table 1.

**Table 1:** Mentorship program indicators

**Indicator**	
**Trainees**	(*n* = 80)
Mean age (years)	30.00 ± 2.19
Gender	
Male	5 (6.25%)
Female	75 (93.75%)
**Meetings**	(*n* = 303)
Mean Duration (mins)	20.08 ± 9.50
Mean number of meetings per trainee	3.88 ± 2.31
**Type of meeting**	
1. Face to Face	25 (8.3%)
2. Phone	188 (62%)
3. Virtual	90 (29.7%)
**Mentors**	(*n* = 16)
Ratio of trainees per mentor	5/1

### Challenges identified from the mentorship program and related interventions

**Fig. 2:**
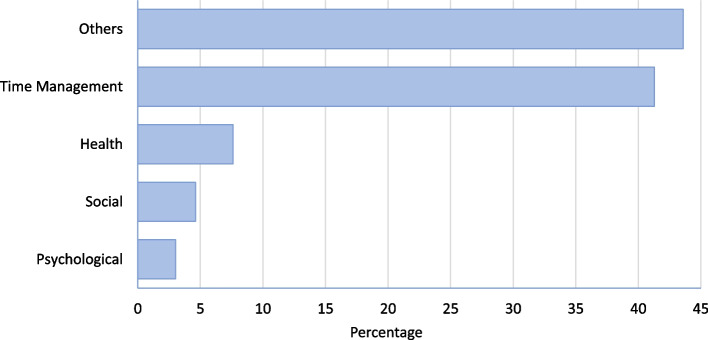
Description of the challenges summarized from mentorship sessions in the quantitative study

The analysis of the quantitative data related to the challenges reported by mentors revealed that time management was the most reported issue affecting the progress of the trainees (41.3%), followed by health-related (7.6%), social (4.6%), and psychological issues (3%). Interestingly, 43.6% of other types of challenges were reported, and they are detailed in the qualitative part of the study. Figure 2 shows the presentation of different challenges as reported by mentors.

The qualitative part of the study permitted to obtain very important information from struggling trainees, particularly sensitive information such as health and mental or psychosocial related issues. We extracted four main themes, such as challenges, and related subthemes data from the mentoring meeting as well as the academic committee face-to-face meeting reports. The main themes are related to trainees, training setting, E-portfolio, and COVID-19 challenges and are detailed below.

### Trainees’ related challenges

Trainee-related challenges included five main sub-themes: health-related, psychological, social, learning style, and time management.

Some of the trainees had chronic medical problems such as multiple sclerosis, systemic lupus erythematosus, sickle cell disease, and migraine as expressed by other trainees “ My migraine attacks are occurring more frequently and it affects my study” and “ “ I am afraid that my admission in the hospital will affect my training and need guidance on how to catch up”.

In addition, others suffered from pre-existing mental health problems such as depression and anxiety as expressed “. Being diagnosed with medical and mental health problems and exposed to the additional stress caused by the intensive nature of the curriculum in the COVID-19 pandemic context, some trainees had low self-confidence and psychological challenges in their capacity to pursue the program as expressed by one of the trainees: “ I am not sure if I can cover all the training requirements, and study, and feel lost”.

Some other trainees were facing some social challenges such as the death of a close relative, the birth of a new child, and being a caregiver of young children, as expressed by a trainee “ I feel that my study progress is slow since I need to manage between my study in the program and taking care of my two little daughters and their requirements”.

The intensive character of the program generated serious challenges to some trainees related to time management and a fair balance between the program requirements and their other life aspects “ I am not sure if I am studying correctly….it is difficult to manage my time”. A group of trainees struggled to adapt their learning style to the primary care approach and setting which favors a learning based on clinical presentations. The latter challenge was very difficult to overcome particularly for freshly graduated trainees who are still influenced by the undergraduate learning mindsets.

Trainees facing such challenges were considered by the academic committee at higher risk and required intensive mentoring. They were entitled to close monitoring to overcome a stressful period.

### Training setting challenges

The training takes place in the regular primary care setting in which the trainers are primary care physicians assigned to train besides managing their scheduled patients’ lists. This situation permitted to immerse the trainees in a real context of family practice but created the challenge of trainers' dedication toward training and facing the problem of unavailability of training rooms in some health centers on some occasions busy trainers who were challenged to manage their role as trainers and other duties as stated by trainees sometimes: “There are no vacant consultation rooms and we alternate with the trainers in conducting the consultation…” and “The trainer is not available all the time to provide detailed feedback on my performance since she is busy as well with other tasks”.

The study also permitted, through probing, “deviant cases” such as students suffering from mental health or chronic diseases that required special care such as a placement in a more psychologically- safe training environment (a more compassionate clinical trainer). Some other unexpected findings emerged from the probing such as the rejection of an experienced trainer because of a perceived “autocratic” vertical approach in training as well as loading the trainees by contents rather than best approaches in learning as expressed “My trainer is treating us in a very rigid way which makes me feel uncomfortable in my training and doubt if I’m not doing well”. Surprisingly, the same trainer was highly appreciated by other trainees. These conflicting patterns discovered through in-depth interviews might reflect different personality traits and cultural frameworks in the study group.

### E-portfolio-related challenges

E-portfolio identified challenges were the suboptimal entry of cases and procedures encountered by some trainees and poor quality of documentation. The trainees did not consider electronic documentation of the daily activities as a high priority. It was usually left for a later time in the week, which increased the recall bias and incompleteness of information as expressed by a trainee “ Sometimes, I do not have the time to document in the E-portfolio and I have a lot of pending work related to my E-portfolio….I try to do it in the weakened”. This difficulty was reduced through feedback meetings and the improvement of E-portfolio forms in the Moodle platform.

### COVID-19 related challenges

The program started during the first period of the COVID-19 pandemic when the social distancing precautions and regulations that included primary health care centers were strictly enforced. This resulted in a limitation in terms of the number and variety of clinical cases encountered especially in preventive and non-communicable diseases as expressed by a trainee “ There is a very low flow of patients and I am concerned that it will affect my learning”. In addition, we faced a gap in training for minor surgical procedures including those in primary care settings as expressed by a trainee “ I was not trained in any minor surgical procedures during this rotation….all minor non-urgent procedures were withheld due to the COVID-19 regulations”.

**Fig. 3:**
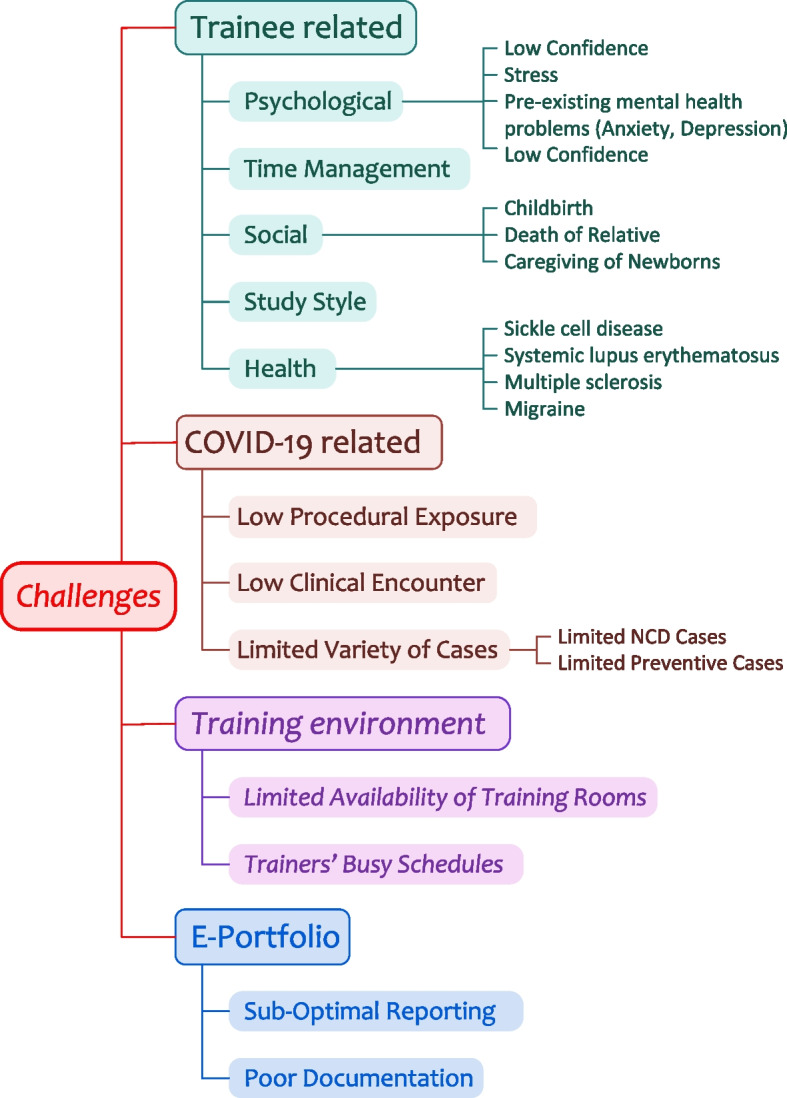
Main Themes and sub-themes of challenges identified from the qualitative contents of the mentoring reports

Figure 3 illustrates themes and sub-themes for these challenges.

Triangulating the information from the mixed method approach permitted the CMFM program academic committee to obtain a comprehensive situation analysis of the progress of every trainee. Consequently, it allowed the timely implementation of relevant personalized corrective measures by the academic committee, to support the trainees and pursue their normal academic progress. These interventions are described in Table 2.

**Table 2:** Identified themes of challenges and implemented interventions by the academic committee

Challenges	Interventions
**Trainee-related** (health, psychological, social, time management, and learning style)	- Psychological support and stress management advice
	- Recommendation and facilitation of follow-up with medical/mental health care physicians
	- Distance learning toolkits when trainees are absent from training
	- Group and individualized coaching on time management and learning approaches by experts in medical education
**Training setting** (trainers’ busy schedule and limited availability of training rooms)	- Allocating one hour protected per day for training by clinical trainers
	- Selection of centers with more available rooms for training
**COVID-19 related** (disruption of training for affected or contacts, limited number and variety of cases encountered, and minor procedures)	- Case-based discussions
	- Case-simulation
	- Integration of practical skills and minor procedures sessions in the simulation-training center on campus
**E-portfolio related** (sub-optimal documentation and poor quality of documentation)	- Longitudinal training and meetings with trainees to guide and receive feedback
	- Adaptation of E-portfolio to be more user-friendly and less time-consuming

### Evaluation of the performance of the mentorship program

Out of the 80 trainees, 12 (15%) were identified as high-risk trainees, 6 of the 12 (50% of the high-risk) graduated on time while the remaining had to repeat some courses to pass the exit assessment and obtain the master's degree.

When we consider the mean global GPA of high-risk trainees (*n* = 12) during year one, it was 3.22 (SE = 0.16) versus 3.41 (SE = 0.05) for low-risk trainees (*n* = 68), (*P* = 0.33). During year two the mean GPA for the low-risk trainees was 3.29 (SE = 0.07) versus 3.06 (SE = 0.03) for high-risk trainees, (*P* = 0.04). Interestingly, the overall mean cumulative GPA was 3.35 (SE = 0.03) for the low-risk trainees, versus 3.14 (SE = 0.08) for the high-risk trainees, (*P* = 0.043). These findings suggest that trainees are mainly challenged in the second year, but the discrepancy between the high-risk and low-risk trainees significantly reduced at the final cumulative GPA implying the effectiveness of the corrective action plans resulting from the mentorship program.

The academic committee considered the GPA of trainees as one of the main outcomes reflecting the effect of monitoring, including the mentorship program, of progress of trainees, and the implementation of timely corrective actions. When we consider the mean GPA trends over time and cohorts, we realize that the mean GPA in year 1 (GPA 1) for Cohort 1 (40 trainees) was 3.43 (SE = 0.06) versus 3.45 (SE = 0.07) for Cohort 2 (40 trainees) (*P* = 0.022). On the other hand, the mean GPA in year 2 (GPA2) for cohort 1 was 3.18 (SE = 0.04) versus 3.33 (SE = 0.04) for cohort 2, (*P* = 0.02). Despite this slight significant difference, the two cohorts achieved equivalent successful GPAs in both years when we consider the cut-off of a minimum of 3 out of 4 required for the master’s degree. This was corroborated by the final cumulative mean GPA of 3.30 (SE = 0.03), versus 3.34 (SE = 0.05) for cohorts 1 and 2 respectively, (*P* = 0.40). This reflects the stability of the performance of the program over time and cohorts.

## Discussion

We conducted a longitudinal study using mixed methods to describe the implementation of a mentorship program and evaluate its effectiveness in the context of an intensive two-year CMFM curriculum that started during the COVID-19 pandemic. We obtained a real-time comprehensive evaluation of the progress of trainees through parsimonious quantitative indicators and qualitative analysis of challenges they are facing, which was instrumental in designing real-time, personalized corrective actions. The mentorship program proved to be effective for the smooth academic progress of trainees and reduced the risk of failure in graduation. It supported trainees’ overall well-being while maintaining work-life balance and minimizing burnout.

The CMFM mentorship program helped to ensure that the trainees’ progress was meeting curriculum standards and certain key performance indicators related to the training. Mentors provided a longitudinal and holistic evaluation of training that helped to bridge the gap between theoretical learning and clinical practice and suggested recommendations to ensure progress and attainment of appropriate skills and program standards on time.

Our study confirmed that the COVID-19 pandemic threatened postgraduate medical trainees’ academic advancement by constraining opportunities for knowledge and skill acquisition, scholar productivity, and networking. On the other hand, the pandemic has created new opportunities. The exerted challenges of the pandemic-era circumstances required extra efforts and innovative solutions aimed at enhancing trainees’ academic progress while supporting work-life balance [34].

In agreement with others, the consistent approach to mentoring, oversight continuous monitoring, and feedback facilitated oversight and regulation of the mentoring processes [35]. Findings in the literature highlight that several health science faculties could avoid mentoring due to numerous factors including the lack of knowledge about the mentoring process, lack of confidence, and the fear of managing ‘challenging situations’, including problems of a personal nature [36]. Indeed, to standardize the mentorship program and facilitate its implementation, the CMFM program committee provided a mentoring program guide and workshops targeting both mentors and mentees before implementation. Our findings confirm the importance of these preparatory aspects before launching the mentorship program. In addition, mentors attended several longitudinal workshops while the program was running to build their capacities as mentors, receive their feedback, and provide them with guidance and support. Similarly, we conducted periodic meetings with the trainees to identify any issue affecting the mentoring process and relationship that needs timely interventions. Trainees who were unsatisfied or faced any challenges related to the mentor–mentee relationship were assigned to other mentors to ensure the accomplishment of outcomes through academic and personal support.

One of the innovative and strong aspects related to the CMFM mentorship program is structuring an electronic mentoring meeting report with a mixed structure in data collection that helped to harmonize mentor–mentee discussions during the meetings without compromising the specific needs of trainees. Since mentors are composed of family physician consultants and some of them are involved as trainers, we expected the possibility for mentors to focus more on trainee-related issues compared to other factors. Indeed, mentors with integrated physician and mentor identities can embrace liminality and develop as mentors, this was addressed through guidance and support [37]. This tendency is reflected in the frequency of challenges listed in the quantitative study. However, this was fixed by the findings of the qualitative section in the form that permitted to grasp a richer understanding of the trainees' global progress and constraints for pertinent and customized corrective actions as detailed in the findings. This mixed format provided a standardized structure without compromising flexibility in areas of discussion, rapid interventions, and follow-up [2]. Another strength related to the CMFM mentorship program is the formal one-to-one mentoring providing a safe and non-judgmental environment for discussions and personalized advice and guidance. This contrasts with other mentorship programs where the mentor conducts group meetings with mentees [2, 38]. The various options, face-to-face, virtual, and phone calls, for mentoring meetings, eased continuity in meetings, especially during the period of COVID-19. Another strength of our mentorship program is a cascade of checkpoints and interventions at the level of mentors, students’ feedback meetings, and academic committee oversight particularly for trainees at high risk. This permitted a large consensus about corrective plans and strong governance of a complex and intensive program.

The identified challenges through the CMFM mentoring program are consistent with those reported in other postgraduate clinical training programs. The major reduction in the volume of inpatients and outpatients encountered during the pandemic affected the number and diversity of clinical exposure and mitigated drastically the opportunities for trainees to perform physical examinations and essential procedures, which can be mastered mostly during clinical training [39–41]. It was reported similarly in other studies, that the ongoing pandemic has added new stressors while aggravating the existing ones for students and trainees [40, 42]. On the contrary, the pandemic has created new opportunities for the CMFM program academic committee, trainers, and mentors, to sustain and enhance training outcomes. Trainers had more time dedicated to interactions and discussions around selected clinical cases leading to deep clinical learning and high self-confidence. The role of teleconsultation, underutilized in the pre-COVID era, was integrated to ensure continuity of healthcare delivery during the current pandemic by the healthcare system, which offered an opportunity for integrating training on telemedicine and teleconsultation. These skills are nowadays necessary to continue with safe, high-quality delivery of services and increase this modality of care integration in healthcare systems [43]. Another innovative aspect of the CMFM program was the utilization of trainees' E-portfolio entries related to the clinical cases encountered, skills and procedures that they were exposed to, and mentoring meeting reports to identify gaps related to clinical exposure, mainly during the COVID-19 pandemic. This allowed the implementation of pioneering interventions such as engaging learners in experiences that simulate reality and compensate for cases of fewer encounters. Trainers with the help of program administrators also integrated simulated scenarios followed by constructive feedback discussions during clinical training and on weekly educational activities. We also highlight the big added value of on-campus skills and procedures training in the Medical Skills and Simulation Center using high-fidelity mannequins. Simulation is a useful modality to supplement training in real clinical situations because it allows control over the sequence of tasks offered to learners, provides opportunities to offer support and guidance to learners, prevents unsafe and dangerous situations, and creates tasks that rarely occur in the real world. It is also an excellent form that supports inter-professional and communication skills education [44]. We also integrated team-based learning to promote active learning and enhance inter-professional skills development [45]. In addition, trainees whose training was disrupted for any reason (birth of a new child, contact with COVID-19 cases, health-related), and whose situation allowed distance learning were provided with a distance learning toolkit containing clinical scenarios followed by smart questions and recommended online courses related to the ongoing clinical rotation. They received more intense mentorship and administrative support to overcome their challenges.

The monitoring of smart few KPIs during the mentorship program permitted, in our context, the early detection of struggling trainees before the summative exam, allowing timely corrective actions to be implemented.

The CMFM program is system-centered and integrated into primary health care, which increased the ownership; by health authorities and preparedness to facilitate any action needed to better prepare the training environment for an optimized outcome and increased the recruitment of graduated trainees. The provision of a protected time for the trainers to discussions around clinical cases of high educational value and the availability of independent consultation lists and rooms under the supervision of the trainers was particularly instrumental in facilitating deep learning and enhancing the level of self-confidence, safety of trainees and their immersion in a real context of practice.

The learning environment, an important dimension in our mentorship program, was adapted to promote changes in students' thinking strategies as well as their development as flexible, reflective learners [46]. These endeavors require support from mentors and program administrators with rigorous expectations and good facilitation skills. The mentorship program was successful and effective, particularly when coupled with longitudinal meetings and feedback from trainees, trainers, and mentors to get the best comprehensive analysis of the situation and to implement the most appropriate intervention plans. Students’ voices and perspectives as important stakeholders in the process of learning are essential to providing emotional and cognitive support that enhances learning and prevents burnout [46].

In addition to the mentioned benefits for all trainees, the mentorship program identified 12 trainees (5%) at high risk for failure. Six of them (50%) achieved high scores and obtained their degrees at the end of the program due to early identification, extensive follow-up, and support by the program’s committee. Five out of the remaining six trainees obtained their degrees after repeating a few courses to ensure the needed level of safety and competency. Only one trainee out of the two cohorts (80 trainees) graduated with a diploma because the final GPA was less than the threshold of 3/4 as required by AGU regulations. On the other hand, when we consider the overall GPA of high-risk trainees and the rest of the cohort, the difference is not significant which reflects the effectiveness of corrective plans. In addition, analysis of GPA through years and cohorts demonstrates the stability of the performance of the CMFM program over time, partly due to the mentorship program.

This study highlights the importance of a mentorship program in supporting and monitoring postgraduate training in family medicine practice. The lessons learned here lay the foundation for the design of formal and contextualized mentorship programs that align with the training context and curriculum, and the importance of engaging both mentors and mentees in the mentoring process through several aspects including training, guidance, and longitudinal monitoring. All of these aspects, in addition to setting specific key performance indicators, are essential for sustainability, robustness, and meeting intended outcomes. Our recommendations are in alignment with the literature findings regarding the value of designing a customized, holistic, longitudinal mentoring assessment tool in facilitating mentoring and providing timely and specific support to the evolving needs of mentees as they develop their clinical competencies [47]. In addition to the importance of institutional support, adapting programs to local needs and resources, and mentors’ engagement and training for sustainability and performance [48].

Mentorship programs can be instrumental, as we found in this study, in identifying challenges associated with postgraduate clinical training and executing promptly corrective measures. These challenges can be trainee-related (time management, study style, and physical, mental, and social well-being issues), training-setting-related, and implementation phase-related (COVID-19 pandemic in our situation). Mentorship was reported to be positively associated with specific academic performance, attitudes, and minimizing psychological stress [49]. Addressing these challenges and facilitating identification by triangulating findings from different stakeholders supported the timely implementation of appropriate interventions and the optimization of results. The mentorship program has proven to be beneficial in ensuring trainees' smooth academic development and lowering the risks of failure to graduate. It improved trainees' overall well-being while also promoting work-life balance and reducing burnout.

The CMFM program was a success story, in our hands, due to the inclusiveness of all stakeholders and the robustness of design, process, and monitoring despite the constraints of the COVID-19 pandemic. To the best of our knowledge, the format of the contextualized formal mentoring program and the mixed-method approach as well as the multiple levels of oversight are novel in this study. Despite its originality and the significance of its findings, this study has some limitations. The mentors’ reports might be prone to subjectivity. The COVID-19 pandemic and other confounders might affect trainees’ performance represented in GPA. However, this is out of the scope of this study. As a future perspective, more detailed qualitative studies targeting mentees and mentors probing their experience are highly recommended, particularly after the COVID-19 pandemic. Evaluating the level of satisfaction and the mentoring experience from their perspectives will provide another insight that we have not formally measured in the current study. 

## Conclusion

A mentorship program implemented in the CMFM program of Arabian Gulf University integrated key performance indicators extracted from a parsimonious e-portfolio and mixed data from mentorship forms as well as periodic face-to-face meetings with different stakeholders. Triangulating longitudinal information using mixed methods design and analyzing at multiple levels permitted timely personalized pertinent interventions.

## Data Availability

Data will be made available by the corresponding author, without undue reservation once requested.

## Abbreviations

AGU: Arabian Gulf University
CMFM: Clinical Master in Family Medicine Program
COVID-19: Coronavirus disease of 2019
GPA: Grade point average
